# A review of the microtremor horizontal-to-vertical spectral ratio (MHVSR) method

**DOI:** 10.1007/s10950-021-10062-9

**Published:** 2022-03-16

**Authors:** S. Molnar, A. Sirohey, J. Assaf, P.-Y. Bard, S. Castellaro, C. Cornou, B. Cox, B. Guillier, B. Hassani, H. Kawase, S. Matsushima, F. J. Sánchez-Sesma, A. Yong

**Affiliations:** 1 University of Western Ontario, London, ON Canada; 2 Université Grenoble Alpes, Université Savoie Mont Blanc, CNRS, IRD, UGE, ISTerre, Grenoble, France; 3 Dipartimento Di Fisica E Astronomia, Alma Mater Studiorum, Università Di Bologna, Bologna, Italy; 4 Utah State University, Logan, UT USA; 5 BC Hydro, Burnaby, Canada; 6 Disaster Prevention Research Institute, Kyoto University, Uji, Kyoto Japan; 7 Instituto de Ingeniería, Universidad Nacional Autónoma de México, CDMX, Circuito Escolar s/n, Ciudad Universitaria, 04510 Coyoacán, Mexico; 8 US Geological Survey, Pasadena, CA USA

**Keywords:** COSMOS Guidelines, Best practices, Earthquake site effects, Site amplification, Microtremor, Spectral ratio, HVSR, MHVSR, Nakamura method

## Abstract

The single-station microtremor horizontal-to-vertical spectral ratio (MHVSR) method was initially proposed to retrieve the site amplification function and its resonance frequencies produced by unconsolidated sediments overlying high-velocity bedrock. Presently, MHVSR measurements are predominantly conducted to obtain an estimate of the fundamental site frequency at sites where a strong subsurface impedance contrast exists. Of the earthquake site characterization methods presented in this special issue, the MHVSR method is the furthest behind in terms of consensus towards standardized guidelines and commercial use. The greatest challenges to an international standardization of MHVSR acquisition and analysis are (1) the *what* — the underlying composition of the microtremor wavefield is site-dependent, and thus, the appropriate theoretical (forward) model for inversion is still debated; and (2) the *how* — many factors and options are involved in the data acquisition, processing, and interpretation stages. This paper reviews briefly a historical development of the MHVSR technique and the physical basis of an MHVSR (the *what*). We then summarize recommendations for MHVSR acquisition and analysis (the *how*). Specific sections address MHVSR interpretation and uncertainty assessment.

## Introduction

The single-station microtremor horizontal-to-vertical spectral ratio (MHVSR) method involves recording seismic ambient noise, also referred to as microtremors or ambient vibrations, with a single three-component seismometer at a location of interest and calculating the (average) ratio of the horizontal-to-vertical Fourier amplitude spectra (FAS). Earthquake site effects are commonly quantified in terms of the vertically propagating, horizontally polarized shear-wave (SH wave) transfer function. For the simple case of a uniform sedimentary viscoelastic layer of thickness, *h*, with shear-wave velocity, *Vs*, overlying viscoelastic bedrock, resonance frequencies (*f*_*n*_) of the SH wave transfer function occur at

$${f}_{n}=\left(2n+1\right)\frac{Vs}{4h}, \left(n=0, 1, 2, \dots \right).$$

Empirical evidence from sites with measured *V*_*S*_ profiles down to bedrock and from small-strain earthquake recordings has shown that the lowest frequency MHVSR peak occurs at the fundamental mode resonance frequency (*f*_0_) of a soil layer over an elastic half-space from SH wave motion (e.g., Field and Jacob 1993; Lermo and Chavez-Garcia 1994a; Bonilla et al. 1997; Bour et al. 1998; Bard 1999; Fäh et al. 2001; Wollery and Street 2002; Molnar and Cassidy 2006; Haghshenas et al. 2008). To determine varying velocities in the soil layering, the formulae by Dobry et al. (1976) and completed in the review of Urzúa et al. (2017) can be useful.

These observations were the essence of the rapid rise in popularity of the MHVSR method where a single tri-axial seismometer, placed on the ground and left to record microtremors for tens of minutes to an hour, can provide a reliable estimate of the site’s fundamental frequency, i.e., *f*_0HV_ is a measure of *f*_0_. Although the use of the MHVSR method has proliferated, standardization of the technique remains unaddressed outside of the Site Effects Assessment Using Ambient Excitations’ (SESAME) project (Bard et al. 2008) and its MHVSR guidelines (SESAME 2004). Notable paper compilations documenting advances and applications of the MHVSR method for soil and building characterization appear in Mucciarelli et al. (2009) and seismic microzonation in Roca and Olivera (2001). The MHVSR method is documented in Vs measurement guidelines for Canadian seismic site characterization in soil and rock (Perret 2015). The current status towards international guidelines of the MHVSR method as part of the COSMOS International Guidelines on Applying Non-Invasive Geophysical Methods for Characterizing Seismic Site Conditions initiative was summarized in Molnar et al. (2018). This paper supersedes Molnar et al. (2018).

The greatest challenges to an international standardization of MHVSR acquisition and analysis are (1) the *what* — the underlying composition of the microtremor wavefield is site-dependent, and the appropriate interpretation of the MHVSR curve and/or theoretical (forward) model for inversion are therefore still debated; and (2) the *how* — many factors and options are involved in the data acquisition, processing, and interpretation stages. This paper briefly reviews the historical development of the MHVSR technique and the physical basis of an MHVSR (the *what*). We then summarize recommendations for MHVSR acquisition and analysis (the *how*). Specific sections address MHVSR interpretation and uncertainty assessment.

### Principles of technique

Seismic ambient vibration methods measure background seismic noise to assess the elastic properties of the earth’s subsurface, in particular, shear-wave seismic velocities, which are directly related to shear moduli. Seismic noise is defined as the ambient vibration of the earth’s surface, generated by the combination of low frequency (< ~ 1 Hz) natural microseisms and higher frequency (> ~ 1 Hz) anthropogenic microtremors (e.g., Bonnefoy-Claudet et al. 2006; Landès et al. 2010). Notable opportunities to better differentiate natural and anthropogenic seismic noise include increasing growth worldwide of wind turbine facilities (e.g., Edwards 2015; Marcillo and Carmichael 2018) and lockdown measures related to the recent COVID-19 global pandemic (e.g., Lecocq et al. 2020). This background noise is a mixture of various seismic wave phases, which contains information on the sources and transmission paths of the waves, and subsurface structure (Okada and Suto 2003). Most anthropogenic seismic noise sources and some natural sources (e.g., wind and ocean waves) are located close to the surface of the earth, which leads to energy mostly released as surface waves. Even for subsurface sources, if the source is located at large distances, surface waves are the dominant component of the microtremor wavefield (below 1 Hz) because their geometric attenuation is much lower than that of body waves (Socco and Strobbia 2004). A common assumption is that, at a distance of more than one wavelength from the source, the microtremor wavefield (generally above 1 Hz) is dominated by surface waves (Arai and Tokimatsu 2005). It is not possible to isolate every wave from microtremor recording at a single station, although it is possible to separate Love waves from Rayleigh waves (Hobiger et al. 2009).

**Fig. 1:**
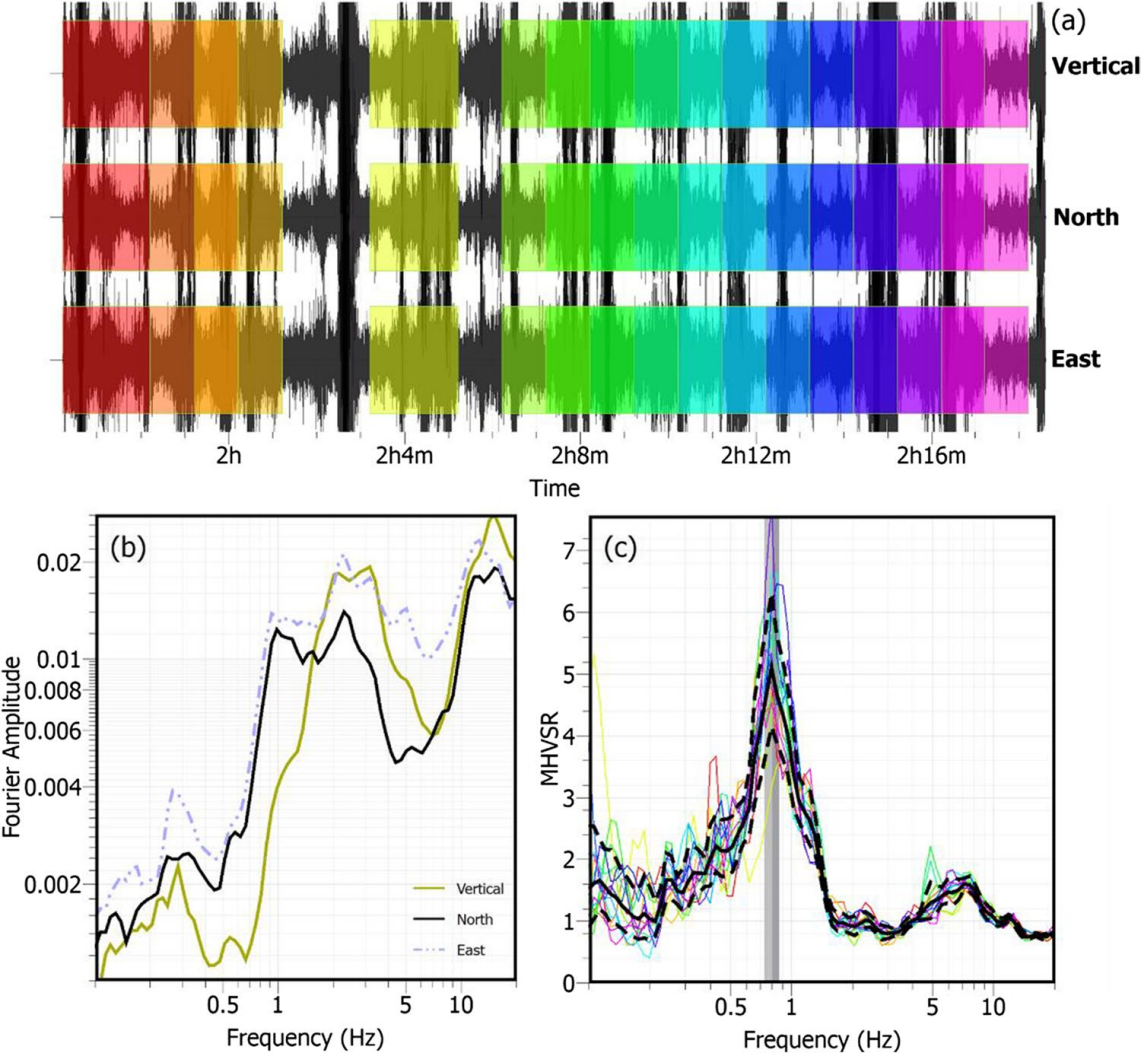
Illustrative example of the MHVSR method. **a** Windowing of microtremor time series. **b** Time window-averaged Fourier amplitude spectra (FAS) for each of the three components. **c** Average horizontal-to-vertical spectral ratio curve (solid black line with standard deviation shown by dashed lines) statistically calculated from each time window’s HVSR (colored lines). The peak frequency of the average HVSR curve and the standard deviation associated with the variability of the peak frequency values from the individual curves is indicated by the vertical gray shading. This example in Metro Vancouver, British Columbia, Canada, is calculated using the Geopsy software package (Wathelet et al. 2020)

The use of microtremors was pioneered by Bertelli (1872) in Italy and then by Omori (1909) in Japan. Aki (1957) proposed to analyze microtremors as a temporal and spatial stochastic process with reference to the nature of wave propagation. The result was a breakthrough, i.e., the well-known spatial autocorrelation (SPAC) method (see Asten and Hayashi, this issue), aimed to retrieve phase velocities of Rayleigh and Love waves. Engineering applications were introduced by Gutenberg (1958) and Kanai and Tanaka (1961). Based on the developments of these individuals, the single-station microtremor approach was adopted by Nogoshi and Igarashi (1970, 1971) in an attempt to understand the characteristics of microtremors and MHVSRs with respect to the site predominant frequency. Nakamura (1989) interpreted that the MHVSR would be the same or similar to the 1D shear-wave transfer function and was the first MHVSR publication in English; see Bonnefoy-Claudet et al. (2006) for a review of early MHVSR literature. Additional pioneering comparisons of MHVSR with spectral ratios calculated from the 1985 Mexico City earthquake recordings were accomplished by Lermo and Chávez-García (1993; 1994a, b). The MHVSR method is an analysis technique (illustrated in Fig. 1) that calculates the ratio of the horizontal-to-vertical FAS derived from microtremor recordings with a three-component seismometer at a specific location. In the previous two decades, Japanese researchers continued to advance and use the MHVSR method (e.g., Horike et al. 2001; Satoh et al. 2001; Arai and Tokimatsu 2004), while it gained popularity and use throughout Europe (SESAME project: Bard et al. 2008), Canada (Molnar and Cassidy 2006; Hunter et al. 2010), New Zealand (Wotherspoon et al. 2015; Vantassel et al. 2018), and South America (Guéguen et al. 1998; Pilz et al. 2009; Leyton et al. 2012). Its use in the USA occurred later but is increasing (Yong et al. 2013; Teague et al. 2018; Stephenson et al. 2019).

Interpretation of the MHVSR curve is complicated by a lack of understanding regarding the precise composition of the microtremor wavefield. Empirical evidence suggests that the MHVSR *f*_0HV_ occurs at, or close to, *f*_0_ if there is a sufficiently strong impedance contrast (e.g., Field and Jacob 1993; Lermo and Chávez-García 1994a, b). Whether the wavefield composition is primarily body waves, surface waves, and/or highly scattered (diffuse) combinations thereof (total wavefield) is still largely debated (e.g., Lachet and Bard 1994; Fäh et al. 2001; Malischewsky and Scherbaum 2004; Bonnefoy-Claudet et al. 2008; Sánchez-Sesma et al. 2011; Lontsi et al. 2015). Studies of microtremors have shown that the contribution of different wave types varies with frequency, from site to site, and that Love waves are often a dominant part of the surface wave component of the microtremor wavefield (e.g., Arai and Tokimatsu 1998; Yamamoto 2000; Köhler et al. 2007; Endrun 2011). Therefore, no single analytical expression exists for all real-world conditions.

## Theoretical background: state of knowledge and ongoing debates

The ambiguity regarding the composition of the microtremor wavefield has made it challenging to theoretically model the MHVSR to infer 1D shear-wave velocity (*V*_*s*_) profiles. The wavefield itself is subject to meteorological, diurnal, and seasonal variations (e.g., Volant et al. 1998; Mucciarelli and Monachesi 1998; Bour et al. 1998; Mucciarelli et al. 2003; Guillier et al. 2007). Over the years, different authors have attempted to explain the MHVSR phenomenology in terms of SH waves (Herak 2008; Nakamura 1989, 1996, 2019) and Rayleigh waves (Lermo and Chavez-Garcia 1994a, b; Fäh et al. 2001; Malischewsky and Scherbaum 2004; Tuan et al. 2011) and by adding the effects of Love waves (Arai and Tokimatsu 2004; van Der Baan 2009). Recent studies consider the role of all waves, the so-called total wavefield approach (Bonnefoy-Claudet et al. 2008; Lunedei and Albarello 2010; Sánchez-Sesma et al. 2011; Lunedei and Malichewsky 2015; García-Jerez et al. 2016; Piña-Flores et al. 2017; Spica et al. 2018).

### Body wave interpretation

Nakamura (1989), in his innovative proposal of the MHVSR, assumed that the microtremor wavefield is primarily composed of S and Rayleigh waves. He proposed that the effects of Rayleigh waves are “eliminated” by considering the spectral ratio. By assuming that the MHVSR at the sediment–bedrock interface is unity, and vertical component motions do not undergo amplification within the near-surface sediments, the MHVSR at the surface can be treated as a “quasitransfer function” from which *f*_0_ and its corresponding amplification factor may be estimated. Nakamura updated his theory by including the contribution of surface waves but claiming their effects are negligible around the fundamental frequency (Nakamura, 1996) and then updated again to include the effects of P waves (Nakamura 2000). Herak (2008) developed an MHVSR modelling algorithm based on this conceptualization of the MHVSR (i.e., the ratio of the S wave to P wave transfer function). Kawase et al. (2011) obtained a similar formula under the diffuse concept for earthquakes with an amplitude correction (scaling) factor between horizontal and vertical components at the seismological bedrock. Nakamura (1989, 1996, 2000, 2008, 2014, 2019) has repeatedly asserted the correspondence between both the peak frequency and amplification factor of the MHVSR and that of the S wave transfer function. Oubaiche et al. (2016) provide an experimental study of the relationship between MHVSR peaks and both the SH wave transfer function and Rayleigh wave ellipticity. They found that the MHVSR *f*_0HV_ matches the peak frequency of the SH wave transfer function but not Rayleigh wave ellipticity. While Nakamura has championed the use of MHVSR for *f*_0_ measurement, claiming that the wavefield composition at *f*_0HV_ is dominated by S waves, the frequency dependence of the wavefield composition implies that the MHVSR cannot be used as a direct proxy for a site’s S wave transfer function. In other words, the shape of the MHVSR is not controlled by S waves alone.

Given the lack of correspondence between not only the shape of the MHVSR curve and the S wave transfer function, but also the amplification factor determined from the two, a method to link the two is desirable. Empirical measurement of “true” earthquake site response (amplification) is accomplished by standard (soil base-to-surface or rock-to-soil) spectral ratios (SSR) of multiple earthquakes from various azimuths (Borcherdt 1970). In practice, SSRs are sometimes challenging to calculate due to a lack of suitable bedrock reference sites and/or earthquakes. As an alternative, the single-station horizontal-to-vertical spectral ratio of earthquake motions (EHVSR) is often used. Due to differences in wavefield composition, strength of ground motions and excitation of higher modes, the frequency-dependent amplification, and spectral shape, as well as estimated resonance frequencies, may vary between earthquake and microtremor spectral ratios. Satoh et al. (2001) performed a comprehensive comparison of SSRs and HVSRs from earthquake P wave, S wave, and coda portions as well as the MHVSR from a separate microtremor recording. They showed that the MHVSR does not coincide with the earthquake SSR and HVSR calculated from the S wave portion, although there was rough agreement in *f*_0_ when it is lower than 1 Hz with an amplitude greater than 3. Horike et al. (2001) also compared earthquake SSRs and HVSRs with the MHVSR at multiple sites and concluded MHVSRs partly reflect but do not agree with S wave SSR amplification. The latest developments in empirical corrections of MHVSR to obtain the site amplification function are presented in Sect. 5.1.

### Surface wave interpretation

As noted previously, many anthropogenic and natural seismic noise sources initiate energy at the ground surface, leading to a common assumption that the microtremor wavefield is composed primarily of surface waves. Nogoshi and Igarashi (1971) compared MHVSR curves with fundamental mode Rayleigh wave ellipticity and concluded that the fundamental mode Rayleigh wave provides the main contribution to the long-period microtremor wavefield. Under the Rayleigh wave ellipticity assumption, the peaks of the MHVSR are related to vanishing of the vertical component amplitude of Rayleigh wave motion near the fundamental site frequency, which occurs when the sense of motion switches from retrograde to prograde or vice versa, in the presence of a large impedance contrast. Malischewsky and Scherbaum (2004) presented an analytical formula to calculate Rayleigh wave ellipticity for a 2-layer model of compressible media. Malischewsky et al. (2008) used this analytical expression to explore Rayleigh wave particle motion as a function of material properties. These studies provide the basis to study two special features of the ellipticity function: the singularity (i.e., maximum) and the zero (i.e., minimum). It has generally been observed that the singularity occurs close to *f*_0_ but only if the impedance contrast is greater than 4 (see Bonnefoy-Claudet et al. 2008). For lower impedance contrasts or low Poisson ratios, the singularity occurs at frequencies between 0.5*f_0_ and 1.5*f_0_, and the minimum is not readily observed.

Many studies have used the relationship between MHVSR and Rayleigh wave ellipticity to invert for layered earth models (e.g., Fäh et al., 2001; Scherbaum et al. 2003; Arai and Tokimatsu 2004; Wathelet et al. 2004; Parolai et al. 2005). Cipta et al. (2018) presented a trans-dimensional Bayesian framework to invert MHVSR curves for 1D profiles, modelling the MHVSR as the fundamental mode Rayleigh wave ellipticity. Studies have shown success inverting MHVSRs for layered earth models only considering the fundamental mode Rayleigh wave (Yamanaka et al. 1994); however, other studies have shown that higher modes make contributions, particularly when low velocity zones are present (Arai and Tokimatsu 2004; Parolai et al. 2005; Rivet et al. 2015; Savage et al. 2013). To improve the results of inversion, Rayleigh wave ellipticity can be extracted from microtremors by using seismic arrays ( Maranò et al. 2017; Poggi and Fäh 2010; Poggi et al. 2012; Wathelet et al. 2018) or single-station methods (Hobiger et al. 2009, 2013).

Studies based on simulating the microtremor wavefield have shown that for impedance contrasts greater than four, MHVSR peaks can be explained by horizontal polarization of the fundamental mode Rayleigh wave coupled with the contribution of the Airy phase of fundamental mode Love wave (e.g., Bonnefoy-Claudet et al. 2008; Konno and Ohmachi 1998; Lunedei and Albarello 2010). The prominence of Love waves in the microtremor wavefield has been asserted by many authors (e.g., Endrun 2011; Köhler et al. 2007; Yamamoto 2000). Konno and Ohmachi (1998) found that if the proportion of Rayleigh waves in the microtremor wavefield is 0.4, the amplitude of the MHVSR peak is close to the S wave amplification factor. Although the peak of the MHVSR is close to the S wave resonance frequency, the MHVSR curve shape is closely related to the fundamental mode Rayleigh wave ellipticity (Arai and Tokimatsu 2000; Konno and Ohmachi 1998). This has been shown to be true not only when Rayleigh waves are dominant in the microtremor wavefield (e.g., Scherbaum et al. 2003), but also in numerical studies that use full wavefield modelling of the microtremor wavefield (Field and Jacob 1993; Lachet and Bard 1994; Lunedei and Albarello 2010).

### Total wavefield interpretation

Lachet and Bard (1994) carried out a numerical simulation of the microtremor wavefield for 15 soil profiles by arranging random amplitude sources uniformly within a given radius from a receiver. The results of their theoretical study indicate that the peak of the MHVSR corresponds well with both the S wave resonance frequency, and the ellipticity peak of the fundamental mode Rayleigh wave. They suggested that the overall MHVSR curve shape is determined by all seismic phases. Field and Jacob (1993) proposed a theoretical formulation to relate the microtremor displacement power spectrum to the Green’s function of the earth’s surface. Lunedei and Albarello (2010) extended this model to include all seismic phases.

By using surface sources and several simple stratigraphic profiles in a numerical study, Bonnefoy-Claudet et al. (2008) checked the correspondence between *f*_0HV_ and *f*_0_. They concluded that depending on the impedance contrast, the MHVSR peak could be explained by Rayleigh wave ellipticity, Love Airy phase, S wave resonance, or some combination thereof. Considering these theoretical and numerical investigations, it is evident that the precise composition of the microtremor wavefield is a complex function of many variables. The most appropriate explanation of the MHVSR phenomena should therefore account for all seismic phases.

Sánchez-Sesma et al. (2011) proposed that microtremors form a diffuse field containing all types of body (P and S) and surface waves (Love and Rayleigh) for which their associated illumination strengths stabilize in fixed proportions. Within this diffuse field assumption (DFA), multiple scattering and its equilibrating effects play a prominent role. The relative power of each seismic state that composes the illumination emerges from the principle of equipartition of energy. Theory asserts that within a diffuse elastic wavefield, the autocorrelation in the frequency domain (the power spectrum), at any point of the medium, is proportional to the imaginary part of the Green’s function for source and receiver at the same point. As average autocorrelations are proportional to average directional energy densities (DED) then, by following Arai and Tokimatsu (2004), one way to assess the MHVSR is the square root of the ratio of the DEDs:

$$MHVSR=\sqrt{\frac{{E}_{1}+{E}_{2}}{{E}_{3}}},$$

where *E*_*#*_ are the DEDs for the horizontal (*E*_*1*_ and *E*_*2*_) and vertical (*E*_*3*_) components. Therefore, DEDs are calculated as the averaged autocorrelation of the three recorded components at a receiver. The DFA allows linking measurements with physical properties of the medium. The MHVSR is modeled in terms of Green’s functions:

$$MHVSR=\sqrt{\frac{Im{G}_{11}+{ImG}_{22}}{{ImG}_{33}}},$$

where *ImG*_*ii*_ is the imaginary part of the Green’s function for the *ith* component for a unit harmonic excitation in the *ith* direction when both source and receiver coincide. For a horizontally layered medium, this approach is straightforward for surface recordings (Sánchez-Sesma et al. 2011; Kawase et al. 2015) and at depth (Lontsi et al. 2015) and even under a water layer in offshore settings (Lontsi et al. 2019). Considering the microtremor wavefield as not systematically diffuse and equipartitioned, Tchawe et al. (2020) proposed to compute MHVSR on the coda part of the microtremor correlations. Sánchez-Sesma (2017) addresses non-uniqueness arising from inverting only MHVSR or by jointly inverting the MHVSR (under the DFA) with fundamental or higher mode surface wave dispersion curves. Wu et al. (2017) propose a simplified full wavefield approach to invert 1D velocity structure directly from the MHVSR on the basis of locked mode approximation. Lateral heterogeneity can be dealt with similarly, but computing Green’s functions becomes computationally expensive (Matsushima et al. 2014, 2017). Some encouraging results are due to Spica et al. (2015, 2017, 2018) and Perton et al. (2017).

The search for indicators of field diffusivity is ongoing. Using empirical recordings from a dense 72 station array, Pilz and Parolai (2014) employed multifractal detrended fluctuation analysis to assess wavefield character ranging from ballistic to diffusive considering varying timescales and seismic intensities. They found the character of motion varies from nearly ballistic to diffusive on frequency-dependent timescales for all materials. A time windowing scheme has been proposed to enhance diffuse properties of the field (Weaver and Yoritomo 2018). Therefore, the corresponding processing must be the same to fully exploit the diffuse field nature. Under certain circumstances, the P and S energy equilibrates, and this process anticipates the emergence of a diffuse regime (not necessarily isotropic), which justifies the interpretation of MHVSR under the DFA (Piña-Flores et al. 2021).

## Microtremor recording

Microtremor data acquisition is relatively simple, requiring a single tri-axial seismometer to record for tens of minutes to hours (i.e., very little equipment and personnel required). Poor choices made by the practitioner during data acquisition have the potential to impact the MHVSR by introducing artificial resonance frequencies (MHVSR peaks) and altering the MHVSR amplitude. The natural site-related resonance frequencies are relatively robust and likely to be obtained even if the data acquisition is not ideal. This latter point combined with the minimal required equipment is the appeal of the MHVSR method. The greatest difficulty is at the interpretation stage; the practitioner should therefore seek to minimize errors during the data acquisition and analysis stages.

There is no international standard related to documenting the metadata associated with each microtremor recording. An example metadata field sheet was generated in MHVSR guidelines of SESAME (2004) and Canada (Perret 2015). The recommended minimum metadata includes equipment serial number(s), acquisition file identifier, material type the sensor is placed on/in, a site photo, and notes of only “anomalous” recording conditions, i.e., the default assumptions that “good” sensor coupling is achieved and observed seismic noise sources (environmental and anthropogenic) will not impact or degrade the MHVSR need not be documented. Any deviation from these default assumptions should be documented. Documenting the equipment used (e.g., seismometer-digitizer pair, if applicable) by their serial number or identifier is beneficial when multiple equipment is available and used over many years or in different campaigns. Field notes and site photos provide direct benefit to MHVSR calculation and interpretation. Documentation of metadata becomes cumbersome as the quantity of microtremor measurements increases into hundreds and thousands of sites. Large quantity microtremor campaigns performed for seismic microzonation mapping, site characterization of a seismic network’s stations, or post-earthquake reconnaissance purposes (e.g., Puglia et al. 2011; Albarello 2011; Molnar et al. 2020; Ladak et al. 2021) may include multiple equipment, practitioners, and/or span multiple years. In such cases, the importance of minimal standardized yet robust metadata of the microtremor measurements and their processing and interpretation increases.

Recommendations on suitable equipment and deployment and acquisition selections provided in the next two sections are based on outcomes of the SESAME project and updated when applicable. The SESAME (2004) guidelines and Koller et al. (2004) provide summaries on their equipment and experimental testing conditions which are each documented fully in SESAME WP02 deliverables D01.02 and D08.02, respectively (http://sesame.geopsy.org/SES_TechnicalDoc.htm, last accessed July 2021). Relevant figures available in those deliverables, and their appendices are not repeated here but are a valuable resource for the beginner.

### Equipment

The most suitable recording instrument is a three-component seismometer (velocimeter) with a noise floor lower than the seismic noise level over the frequency band of interest (i.e., 0.1–25 Hz). Ensuring that the internal noise of the entire acquisition system is much lower than the measured microtremors is the most important factor in selecting appropriate equipment. The natural frequency of the instrument or sensor should be considered with the estimated site conditions. For sites with deeper impedance contrasts, broadband or low-frequency seismometers are recommended, whereas for shallower impedance contrast sites, higher natural frequency seismometers are suitable even if some authors claim that low-frequency peaks can be identified by short-period seismometers (Castellaro and Mulargia 2009a; Chatelain and Guillier 2013; Molnar et al. 2020). In comparison to short-period seismometers, broadband seismometers have a flat instrument response to much lower frequencies but are more difficult to deploy for short-term experiments due to a longer stabilization time and sensitivity to climatic conditions (Guillier et al. 2008). Additional published comparisons between broadband and short-period seismometers and/or between various sensor–digitizer pairs can be found in Castellaro and Mulargia (2009b) and Strollo et al. (2008). Accelerometers with a relatively high intrinsic noise level should generally be avoided (Guillier et al. 2008); use of accelerometers for free-field microtremor measurement is no longer prevalent (e.g., Theodulidis and Bard 1998).

**Fig. 2:**
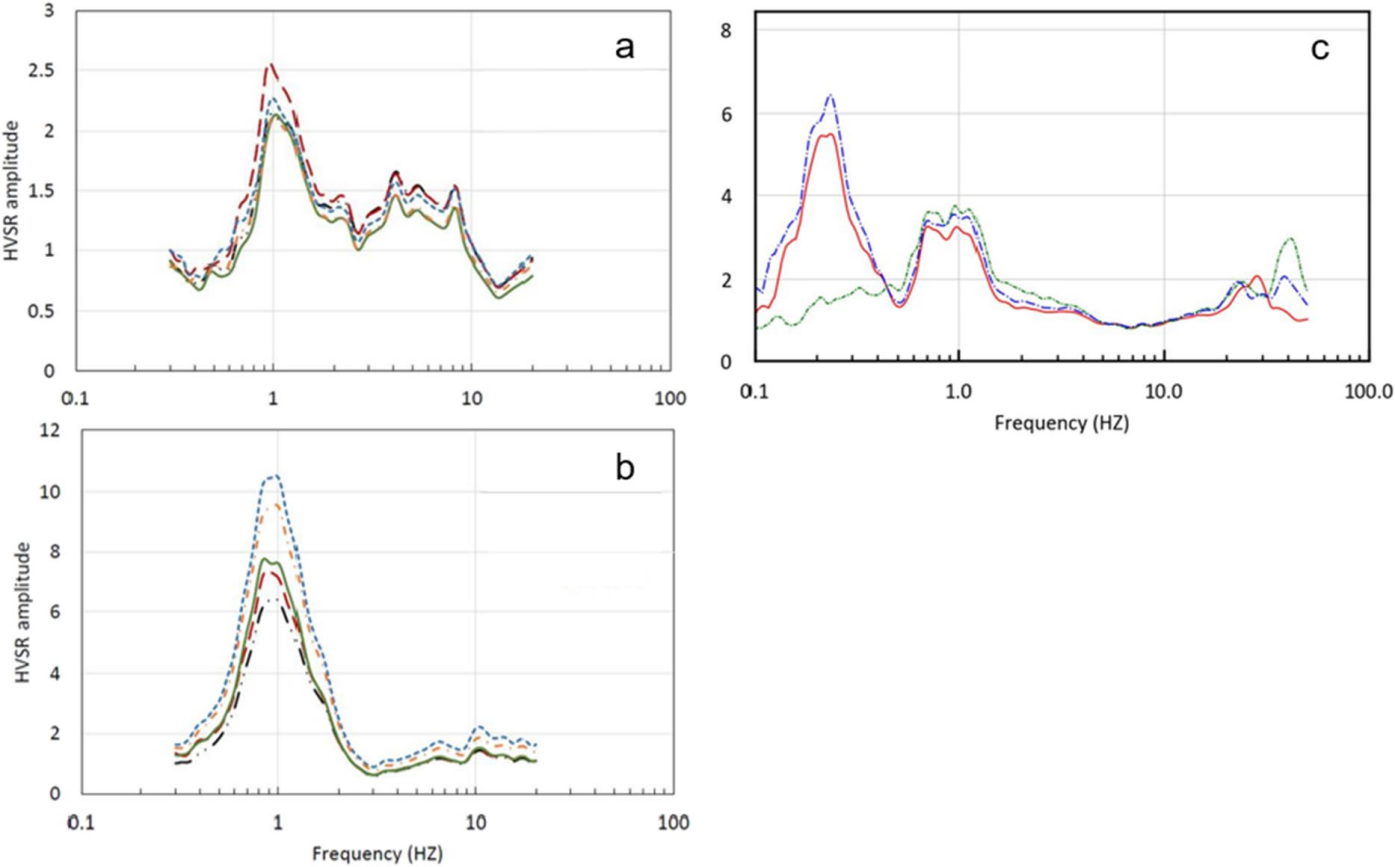
Variability in average MHVSRs depending on equipment. **a**, **b** For a site in Texas, USA, comparison of average MHVSRs from co-located broadband seismometers (red, green lines) and short-period 2 Hz or 5 Hz seismometers (other color lines). **c** For a site on the Fraser River delta, Canada, comparison of average MHVSRs from co-located broadband (red line) and two generations of a short-period seismometer (green line corresponds to older generation compared to blue line)

Figure 2 shows examples where the mean MHVSR obtained from simultaneous recordings using short-period and broadband seismometers immediately beside each other have nearly identical peak frequency values. However, Fig. 2 shows that a very low *f*_0HV_ (0.2–0.3 Hz) can be missed within the equipment noise level when using a lower sensitivity short-period seismometer at a low seismic noise site regardless of recording duration. In terms of MHVSR amplitude, cases of consistency (Fig. 2, 2) and inconsistency (Fig. 2) between equipment types occur even when care in equipment deployment is accomplished (not a sensor coupling issue). Variability in the measured MHVSR amplitude between equipment types at a site is one facet of the caution towards direct inversion of MHVSR curves (discussed further in Sects. 5.2 and 6.2). One can certainly find MHVSR comparison demonstrations like Fig. 2 obtained using 2–3 different sensor–digitizer pairings in single publications from the last two decades, but overall, there has not been a comprehensive or international benchmark testing of equipment since the SESAME project (Guillier et al. 2008) which was focused more on reliable peak frequencies and less on the MHVSR amplitude and its entire spectrum shape. The quantity of seismic equipment developed for MHVSR measurement has also increased significantly since the SESAME project.

It is important to check the noise spectrum at a site to determine the frequency range for which results are valid. If there is not sufficient energy at certain frequencies, this may result in spurious peaks (Albarello 2001) or lack of resolution of peaks. The noise spectrum should also be cross-referenced with the theoretical instrument response spectrum to ensure the correct operation of the instrument. It is most often assumed that the instrument response for all three components is identical; this is not necessarily the case (Guillier et al. 2008). If the instrument response differs for each component and is not removed from the measured motions, this can degrade the resulting MHVSR.

### Deployment and acquisition

**Fig. 3:**
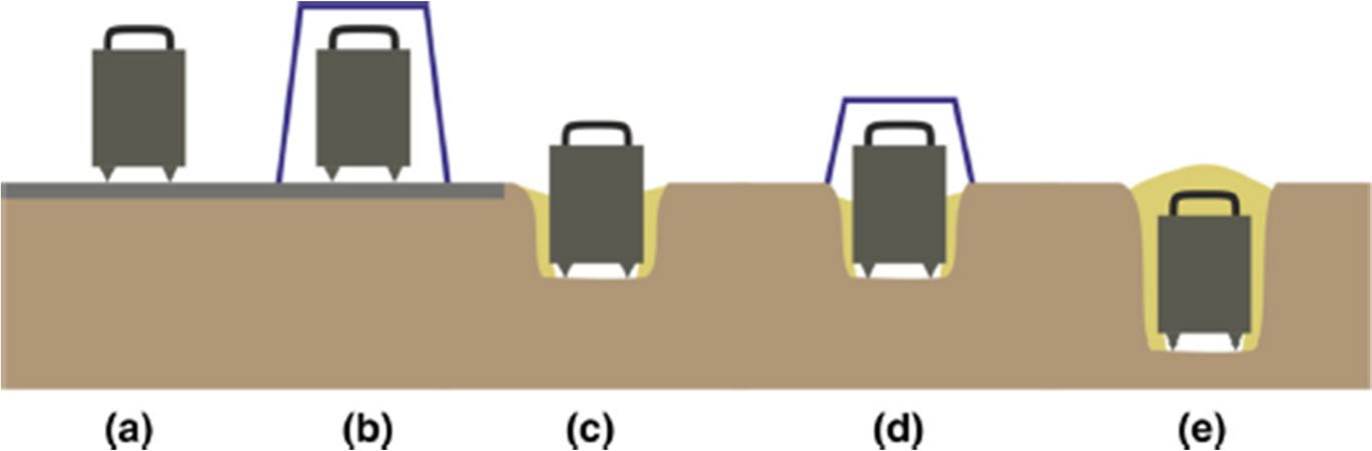
Different possible seismometer deployments for microtremor measurements, ranging from **a** least desirable to **e** most desirable for high-quality data acquisition. Due to the relatively short recording duration, deployments with infill (**c**, **e**) are rare; most deployments are performed according to **a**, **b** or **d** without infill. Adapted from Foti et al. (2018)

Foti et al. (2018) provides an excellent graphic (Fig. 3) that depicts the increasing preference of options for seismometer deployment. The ideal field deployment involves levelling the sensor on/just below the earth’s surface at a location of interest where the surface conditions should be representative of natural free-field ground conditions. Coupling of the sensor to the earth’s surface is crucial. Vegetation should be removed and sensor base or feet inserted firmly into the ground surface with protection from other natural phenomena including temperature fluctuation and wind (cable vibration issues, Mucciarelli et al. 2005) or rain vibrations (Chatelain et al. 2008). Measurements should be avoided during wind and heavy rain. Figure 3 depicts seismometer deployments that can mitigate against climatic factors. For example, coverage of the sensor (e.g., a bucket) is depicted in Fig. 3. A cover can help shade and keep the seismometer cool but can also lead to transmitting wind or rain droplet vibration into the ground due to its surface area. Waiting for a less windy day or use of an umbrella with fewer contact points is often better solutions towards mitigating recording wind and rain vibration. Ideally, a huddle test is accomplished to verify the interoperability of the equipment (i.e., several digitizer–seismometer pairs) but also to verify high-to-low noise sites to assess the dynamics of the digitizer–seismometer pair.

Human-constructed surfaces or pavements should be removed prior to sensor-ground coupling. However, in urbanized settings, this is almost never possible, and most recordings will be collected on human-constructed pavements, e.g., asphalt, concrete, and stone. Measurements on stiff pavements over softer soils creates a velocity inversion in the subsurface which leads to reduction in the MHVSR below unity (deamplification) at frequencies above *f*_0HV_ (Castellaro and Mulargia 2009b; Piña-Flores et al. 2020) but does not impact *f*_0HV_ (Chatelain et al. 2008). When MHVSR deamplification is observed from recordings on natural ground, it is indicative of natural velocity inversions or low velocity zones (Di Giacomo et al. 2005; Castellaro and Mulargia 2009b; Piña-Flores et al. 2020).

In general, any other factor that degrades the sensor-ground coupling or violates the free-field ground condition requirement should be avoided (i.e., synthetic interfaces) (Chatelain et al. 2008), if possible, or minimized. Recording over subsurface cavities or other buried human-constructed materials (e.g., electrical power lines or vents) will alter the MHVSR curve (Chatelain et al. 2008) and are recommended to be avoided; cavities that are shallow and wide violate the free-field requirement. For proximity to urban infrastructure, a general criterion is to offset the recording location by a distance equivalent to the height of the structure (Chatelain et al. 2008; Castellaro and Mulargia 2010); however, this is not always possible. If this criterion must be violated, caution should be exercised when interpreting the results, as the resonant frequency of the structure may show up in the MHVSR recording, notably when recording on the foundation of any built structure and near tall structures or long bridges. In these cases, testing the damping of the *f*_#HV_ is then mandatory and should be strongly less than 1.00 (as in the natural condition). A table of acquisition parameters and their influence on the processed MHVSR is included in Appendix 1 (Fig. 9), adapted from Koller et al. (2004).

The microtremor recording duration must be long enough to show that a statistically stable MHVSR is achieved. The recommended recording duration is inversely proportional to the site’s fundamental frequency and should, with a general criterion, be at least 20(10/*f*_0HV_) (see Sect. 4.1). In addition, the noise wavefield at a site (nearby transient sources), climatic conditions (wind, rain, etc.), and sensitivity of the seismometer used will impact the stability of the MHVSR in time (and standard deviation of the entire curve) and therefore the recording duration. Of these, the controlling factor is the seismometer sensitivity; the less sensitive the seismometer, the less attention required towards climatic and nearby noise sources, but more attention is required towards ability of the sensor to provide reliable recordings of the ambient vibrations rather than instrumental electronic noise. In general, less sensitive sensors will perform better in urbanized environments, as the measurement quality is not degraded by the increased nearby noise sources (traffic, people walking, etc.). In general, climatic and nearby noise sources will impact the contribution of transients to the MHVSR.

During acquisition, the user should exercise judgment to compensate for periods of suspected poor microtremor recording by increasing the measurement duration. In an urban environment, it is recommended to find a location to deploy the seismometer with a buffer zone free from nearby noise sources (busy traffic, people walking, etc.) of 5 m or larger. Traffic is a common concern in highly urbanized environments; however, the MHVSR is not adversely affected even if the sensor is located on natural ground a few meters from a highway (Taniguchi and Sawada 1979; Chatelain et al. 2008). Castellaro and Mulargia (2009b) showed that the low-frequency branch of the MHVSR has increased uncertainty in the presence of a high noise level from a nearby source, while Lunedei and Albarello (2010) confirmed that the presence of nearby sources affects lower and higher frequencies on either side of *f*_0HV_. It is possible that seismic waves from sources closer to the measurement site may only propagate in shallower layers, thus obscuring peaks due to deeper structure (Mihaylov et al. 2016). Overall, to reduce MHVSR variability (standard deviation) at lower or higher frequencies, the user should mitigate climatic factors (improve seismometer deployment (Fig. 3), repeat measurement another day) or nearby noise sources (buffer zone, increased recording duration, tall buildings, and trees), respectively.

Generally, a single microtremor recording per site and its MHVSR calculation are enough to obtain an estimate of *f*_0_, especially if it can be combined with previous earthquake and MHVSR analyses at the same site. At a previously unexplored site, or where artifacts of human origin (e.g., buildings, pavements) are expected, it is recommended to take multiple recordings on different measurement surfaces, at different times of the day or night or on different dates to establish the stability of the MHVSR curve. For regional microzonation studies (e.g., Roca and Oliveira 2001; Moscatelli et al. 2020), measurements should be obtained first at a coarse or large spacing and reduced to a finer scale where rapid variations of *f*_0HV_ are observed.

## MHVSR calculation

The stability and reliability of the calculated MHVSR depend on the processing and interpretation steps as much as on the equipment (acquisition system) and the quality of the in situ acquisition. The general processing steps involved in computing an MHVSR are first outlined below. Then important parameters that are highlighted in the general processing using bold, italicized, and underlined text are discussed in further detail.

The entire microtremor three-component time series is split into several time windows of equal or varying length. ***Window length*** is inversely proportional to minimum frequency; longer time windows should be used for sites with expected low fundamental frequencies (i.e., long fundamental periods; *T*_0_ = 1/*f*_0_). Fourier spectra are computed for each individual tapered time window and ***smoothed***. Each time window should be at least 10 times longer than the estimated fundamental site period, as advised by the SESAME (2004) MHVSR guidelines. After smoothing, the ratio between horizontal and vertical spectra is then performed. Similar individual HVSRs confirm underlying soil homogeneity, while variable HVSRs between individual components may indicate spatially complex, spatially variable subsurface conditions (Guillier et al. 2006; Matsushima et al. 2014, 2017; Vantassel et al. 2018; Cheng et al. 2020). The azimuth dependence of MHVSRs should be checked (e.g., Matsushima et al. 2014, 2017; Cheng et al. 2020) to find indications of two-dimensional (2D) and three-dimensional (3D) site effects including sedimentary basins and surface topography. With additional processing, MHVSR measurements have the potential to provide information on lateral variation of the subsurface, as well as 3D variations (e.g., Hinzen et al. 2004; Grippa et al. 2011; Hallal and Cox 2021), discussed further in Sects. 4.4 and 5.1. The ***average horizontal spectrum*** is valid for use once no azimuthal dependence of the MHVSR is confirmed, i.e., assumptions of 1D site effects can be reasonably accepted. The final average MHVSR for a testing location was historically calculated as the average of all MHVSRs from the spectra of the combined horizontal components of each time window (Nogoshi and Igarashi 1971; Nakamura 1989; Lermo and Chavez-Garcia 1994a, b; Fäh et al. 2001) but is increasingly determined from the averaged spectra from all time windows of each component (Arai and Tokimatsu 2004; Sánchez-Sesma et al. 2011; Lunedei and Albarello 2015; Piña-Flores et al. 2017). Averaging of MHVSRs from all selected time windows reduces variability in the mean MHVSR curve, whereas averaging of each component spectra with time is more appropriate given the diffuse wavefield assumption. The practitioner may manually perform ***window selection*** of the calculated time-averaged MHVSR by removing or “editing out” spurious time windows from the recording. Anti-triggering algorithms can be used to automatically remove time windows based on amplitude (e.g., Wathelet et al. 2020) or frequency content (Cox et al. 2020). Ultimately, it is the practitioner’s choice to limit periods of “bad” seismic noise either during data acquisition (e.g., optimizing the recording of only “good” noise) or during data processing (e.g., remove parts of the time series with “bad” noise).

### Window length

The chosen window length involves a trade-off between spectral resolution and statistically meaningful results. As mentioned, each time window should be at least 10 times longer than the estimated fundamental site period, as advised by the SESAME (2004) MHVSR guidelines. A stable MHVSR will result with 20 time windows or more (Picozzi et al. 2005) with 15–20 windows required to achieve Gaussian statistics. It is common for the authors of this paper to use 30- to 60-s time windows with a minimum of 20 to 50 windows to ensure statistical stability. As such, one should expect to collect a minimum of 15 min of microtremor data. Sites with lower fundamental frequencies (longer fundamental periods) may require a total recording length up to an hour or more to ensure enough time windows are available for reliable processing.

### Window selection

Spurious time windows are identified by the practitioner as statistically inconsistent, often determined by very high amplitudes at the lowest frequencies (e.g., wind effects), low amplitudes (e.g., equipment issues), a wide frequency band of high amplitude noise (e.g., saturated signal from nearby noise source), or knowledge of the source wavefield (e.g., when someone walked past the seismometer). Most software platforms for calculation of MHVSRs have built-in anti-triggering algorithms to remove transient windows based on an STA/LTA algorithm. Mihaylov et al. (2016) developed a routine to separate windows with high- and low-level ambient noise. They observed that the ratios computed with windows of high- vs. low-level ambient noise may deviate, especially in the case where a low-frequency peak is present. Transient removal using wavelet transforms has also been implemented (Vallianatos and Hloupis, 2009). There is debate regarding the impact of including/removing transient time signal. Several authors believe that transients in microtremor records carry information that is highly dependent on the source (Horike 2001; Bard et al. 2008). However, others (e.g., Mucciarelli and Gallipoli 2004) suggest that the non-stationary noise windows should not be removed because they carry information that improves agreement between MHVSR and EHVSR, through introducing more body wave content (e.g., Satoh et al. 2001). Overall, the use of an anti-triggering algorithm, wavelet transform-based removal of transients, or manual selection of windows is best determined on a site-by-site basis. It is worth noting that with a large enough number of windows, transients may have only slight influence on the average calculated MHVSR (Parolai and Galiana-Merino 2006). Considering the lack of agreement regarding how transients affect the calculated MHVSR, frequency-domain window-rejection has been proposed as an alternative. As opposed to considering the time series data, such an approach iteratively rejects time windows corresponding to MHVSR curves that deviate significantly from a statistically defined representative curve (i.e., mean/median). This has the benefit of not only decreasing variance, but also improving data quality when the transient signal is within the same bandwidth as MHVSR peak frequencies (Cox et. al 2020). Automatic window selection has also been accomplished through cluster analysis (D’Alessandro et al. 2016). The cluster analysis extracts self-consistent clusters of MHVSR curves.

### Spectral smoothing

To reduce variance, some smoothing procedures are applied to the individual spectra. Konno and Ohmachi (1998) developed a logarithmic frequency sampled smoothing function to avoid bias in the peak amplitude regardless of frequency. They considered two smoothing functions: a Parzen window with a fixed bandwidth of 0.5 Hz and their proposed logarithmic one. Because the Parzen window has a fixed bandwidth, and due to the commonly employed log-scale of the *x*-axis, as the frequency decreases, the width (number of points) of the Parzen window increases. However, the logarithmic function considers the same number of points, regardless of the frequency (relative bandwidth). The impact of this on the actual MHVSR is that the Parzen window will affect the height of the peak differently depending on the frequency at which it occurs, whereas the logarithmic function does not.

It is recommended to use relative bandwidth filters to smooth MHVSR spectra (Konno and Ohmachi 1998; Parolai et al. 2009). The Konno–Ohmachi smoothing function is most used, with a filter coefficient *b* value of 40. A low *b* value results in very smooth spectra, while a high *b* value results in more erratic spectra. For example, Konno and Omachi (1998) used *b* = 20 in their original work; however, results in their paper clearly show that using a *b* value less than 30 tends to distort the HVSR peaks. Cox et al. (2020) reported achieving stable statistics on the MHVSR peak frequency when using *b* = 40 or 50. Mihaylov et al. (2016) proposed the use of bandpass filters to smooth spectra.

### Combination of horizontal components

The MHVSR is the spectral ratio of horizontal-to-vertical component ground motions. While the vertical component is unambiguously defined, a single “horizontal” component must be defined from the two measured orthogonal components of horizontal motion. As a preliminary assessment of applicability of 1D site effects, MHVSRs of the two orthogonal horizontal components can be calculated (north–south/vertical and east–west/vertical), considering each component of horizontal motion separately. If the two ratios are similar, preliminary confidence is obtained in meeting the assumption of 1D site conditions. It is worth noting similarity of the MHVSR curve in the two measured orthogonal directions does not preclude its variation at other azimuths. MHVSR calculations performed across all azimuths (e.g., Cheng et al. 2020) is a more robust procedure to determine the site’s representative horizontal spectrum, if applicable. Cheng et al. (2020) calculate MHVSRs across all azimuths and combine them statistically to quantify the degree of azimuthal variability in the *f*_0HV_ values. We note that most of the available software (discussed in Appendix 2) may automatically skip confirming azimuthal variability in the MHVSR, displaying only the horizontal average MHVSR but have the option of computing azimuthal MHVSR curves.

The average horizontal spectrum can be defined by considering the arithmetic mean, geometric mean, vector mean, vector summation, quadratic mean, or maximum horizontal value (Albarello and Lunedei 2013). Albarello and Lunedei (2013) concluded that all averaging procedures produce bias; however, by increasing the number of time windows considered, the biases associated with each procedure monotonically decrease rapidly. This is with the exception of the “maximum horizontal value” procedure. Use of the geometric mean is recommended (SESAME 2004; Cox et al. 2020).

## MHVSR interpretation

The SESAME (2004) MHVSR guidelines provide three criteria to identify reliable MHVSR curves (see Appendix 3). If these criteria are not met, adjustment of processing parameters may improve the reliability of the MHVSR curve, as per the first two criteria. Albarello et al. (2011) developed a reliability classification scheme for consistent MHVSR processing that is more conservative than the SESAME guidelines to reduce variability in the extensive processed MHVSR dataset amongst multiple practitioners following the 2009 L’Aquila, Italy, earthquake. Six criteria for trustworthy and interpretable MHVSRs (class A) are based on the Fourier spectra (peaks relate to reduced vertical component amplitude) and sufficient duration leading to robust statistics and ability to evaluate MHVSR consistency with time and azimuth. Albarello et al. (2011) also further subdivides lesser quality MHVSRs; class B corresponds to ambiguous MHVSR curves that are used with caution and interpreted based on nearby MHVSR curves, and class C corresponds to poor quality (uninterpretable) curves that are to be discarded. The described MHVSR reliability classification scheme of Albarello (2011) was also used in determining 193 useable MHVSR curves of 223 Italian strong motion stations (Puglia et al. 2011).

### Selection of MHVSR peak(s)

The primary or most reliable “output” from MHVSR calculation is the lowest frequency peak (*f*_0HV_) which is interpreted to be the site fundamental frequency (*f*_0_). The *f*_0HV_ amplitude, often termed A_0_, is also typically extracted. Higher frequency peaks would be consecutively numbered (*f*_1HV_ and *A*_1_, etc.). We introduce *f*_#HV_ as the most generic term representing a MHVSR peak. A_#HV_ is most often used in a relative sense between MHVSR measurement locations (higher peak amplitudes indicate larger impedance contrasts) and/or as a proxy for strength of impedance contrast(s) but is not a direct measure of soil-to-rock site amplification (discussed further in Sects. 5.2 and 5.3).

**Fig. 4:**
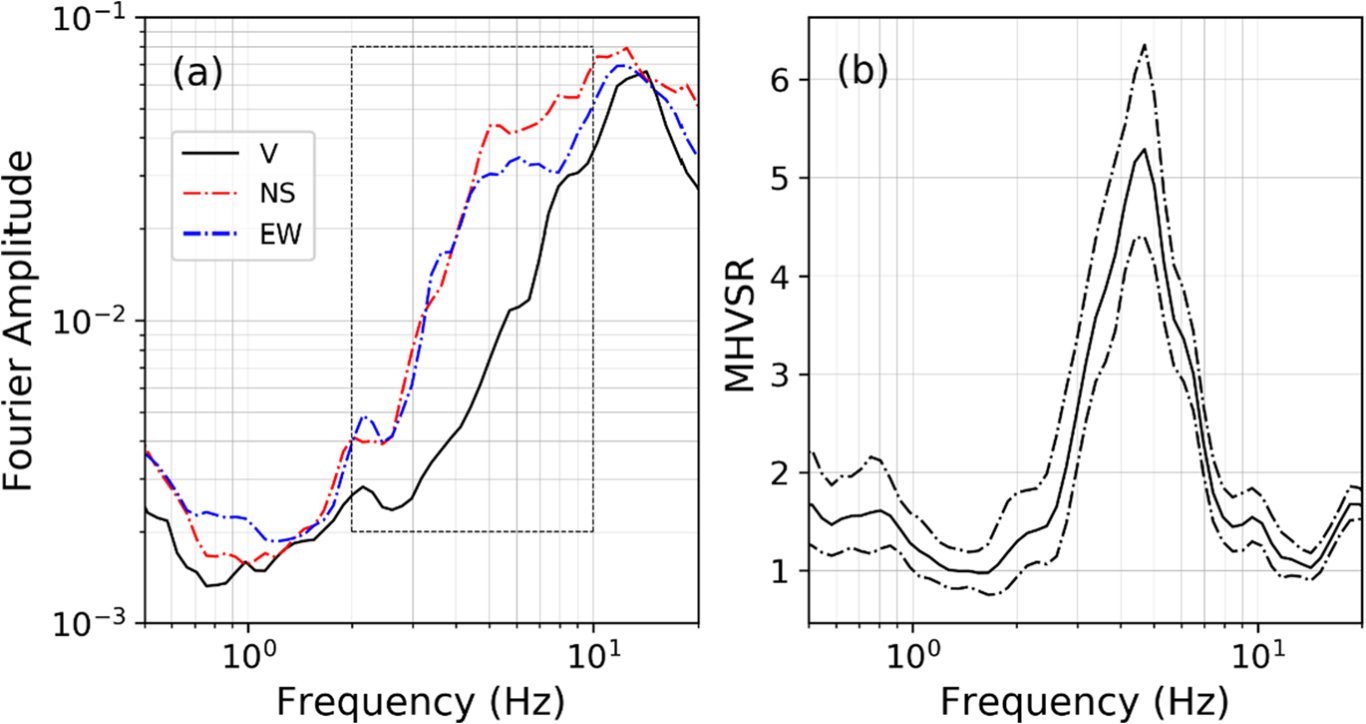
**a** Individual time-averaged Fourier spectrum of each component with typical “eye-shape” (within gray box). **b** Corresponding average MHVSR (solid line) and one standard deviation (dot-dashed lines)

Prior to interpretation, the practitioner must ensure that every peak frequency (*f*_#HV_) has a natural stratigraphic origin. A more qualitative approach is to interpret the calculated MHVSR curve in conjunction with the individual Fourier spectral curves from all three components. A local maximum is expected in the horizontal spectra if the dominant wave type is SH waves. Love waves are indicated by the same feature. MHVSR peaks result from the amplified horizontal component amplitudes. If Rayleigh waves dominate the wavefield, this will result in a trough in the vertical spectral component at the resonant frequency and a peak at 2*f*_0_. In this case, MHVSR peaks exhibit an “eye-shape” in the Fourier spectra (Castellaro and Mulargia 2009b), in which the vertical spectrum amplitude is reduced over a limited bandwidth relative to the horizontal spectra amplitudes. Figure 4 illustrates the “eye-shape” characteristic of a peak caused by Rayleigh waves and stratigraphic origin. Another check involves reprocessing the MHVSR with reduced smoothing. If the peak is of anthropogenic origin, it should become sharper, while this would not be the case for a stratigraphic peak (SESAME 2004). A method to identify anthropogenic origin of a particular frequency in the microtremor time series is the random decrement method (Dunand et al.^ 2002^) which allows the user to know if a specific frequency is damped (natural origin) or sustained (anthropogenic origin). Any peak at a non-damped (< < 1%) frequency will therefore be rejected for interpretation (Dunand et al. 2002; Wathelet et al. 2020). Industrial signals can travel far from their source and can influence the microtremor data even at distances of up to several kilometers from the source particularly at low frequencies (long wavelengths) (Bokelmann and Baisch 1999; Cornou et al. 2004). When the frequency of the anthropogenic signal is approximately the same as that of stratigraphic origin, it is particularly problematic.

The identification of MHVSR peaks is only a straightforward process, either automated or user-selected, when clear peaks are present. The six SESAME criteria for a clear MHVSR peak (Appendix 3, Fig. 10) are well suited for measurements with clear peaks, and each peak is evaluated independently. These six criteria determine whether the peak under consideration clearly “stands out” from the background amplitude level and whether the peak is stable (low standard deviation in amplitude). The greater a MHVSR peak frequency is, the more stringent are the stability conditions for establishing a peak as clear. For all other natural cases (e.g., broad, asymmetrical, or multiple peaks; MHVSR near unity or “flat”), measurements should be assessed on a site-by-site basis. An automated peak-selection process may fail when data is complicated, requiring expert review. Wang (2020) observed that using the SESAME criteria resulted in too many measurements classified as not having clear peaks, which had identifiable peaks based on visual inspection. Wang (2020) modified the SESAME criteria to be more consistent with the subjective assessment of two analysts (Table 1).

**Table 1:** Criteria for picking of clear HVSR peaks (adapted from Wang 2020). Values of Wang (2020) are shown in bold when updated from the SESAME values

Criteria	SESAME	Wang (2020)
Clear 1: *f* ∈ [0.25*f*_#HV_,*f*_#HV_]	A_HVave_(*f*) < 0.5A_#HV_	A_HVave_(*f*) < **0.6A**_#HV_
Clear 2: *f* ∈ [*f*_#HV_, 4*f*_#HV_]	A_HVave_(*f*) < 0.5A_#HV_	A_HVave_(*f*) < **0.6A**_#HV_
Clear 3	A_#HV_ ≥ 2	A_#HV_ ≥ **1.6**
Clear 4: peak of *f*_#HV_ [A_HVave_(*f*)-σ_A_(*f*)]	within [*f*_#HV_ /1.05, 1.05*f*_#HV_]	within [*f*_#HV_ /**1.15**, **1.15***f*_#HV_]
Clear 4: peak of *f*_#HV_ [A_HVave_(*f*) + σ_A_(*f*)]	within [*f*_#HV_ /1.05, 1.05*f*_#HV_]	within [*f*_#HV_ /**1.12**, **1.12***f*_#HV_]
Clear 5: *f*_#HV_ < 0.2 Hz	σ_f_ < 0.25*f*_#HV_	Recommends removal of Clear 5 criteria (barely satisfied)
Clear 5: *f*_#HV_ ∈ [0.2, 0.5] Hz	σ_f_ < 0.20*f*_#HV_	
Clear 5: *f*_#HV_ ∈ [0.5, 1.0] Hz	σ_f_ < 0.15*f*_#HV_	
Clear 5: *f*_#HV_ ∈ [1.0, 2.0] Hz	σ_f_ < 0.10*f*_#HV_	
Clear 5: *f*_#HV_ > 2.0 Hz	σ_f_ < 0.05*f*_#HV_	
Clear 6: *f*_#HV_ < 0.2 Hz	σ_A_ < 3	No change from SESAME
Clear 6: *f*_#HV_ ∈ [0.2, 0.5] Hz	σ_A_ < 2.5	No change from SESAME
Clear 6: *f*_#HV_ ∈ [0.5, 1.0] Hz	σ_A_ < 2	No change from SESAME
Clear 6: *f*_#HV_ ∈ [1.0, 2.0] Hz	σ_A_ < 1.78	No change from SESAME
Clear 6: *f*_#HV_ > 2.0 Hz	σ_A_ < 1.58	No change from SESAME

Rows labeled Clear # represent the #-th condition for a clear peak. *f*_#HV_ is the variable of interest (there could be multiple *f*_#HV_ values in a single curve), A_#HV_ is the amplitude at *f*_#HV_; A_HVave_(*f*) is the amplitude of HVSR mean curve at frequency *f*; σ(*f*) is standard deviation of *f*_#HV_, and σ_A_(*f*) is standard deviation of A_HVave_(*f*)

It is not uncommon to observe double peaks in an MHVSR, sometimes multiple peaks, and the practitioner should review which peaks are of stratigraphic origin (discussed earlier in this section). Secondary peaks are more likely related to a secondary strong impedance contrast, and so forth, and less likely to be higher modes of the fundamental resonant frequency. The lowest frequency peak is caused by the deepest resonator (e.g., overburden/bedrock boundary), and higher frequency peaks are related to contrasts between soil layers within the overburden (Guéguen et al. 2000; Mihaylov et al. 2016). Hunter et al. (2020) observed two significant (*A* > 2) peaks in MHVSR curves which were commonly associated with large velocity contrasts within overburden, either as velocity inversions or as high-velocity inclusion layers. When large velocity variations occurred within the overburden, *f*_0HV_ often appeared to be somewhat attenuated or broadened. However, in other cases, only moderate shape modification was observed. Higher modes are not commonly observed in MHVSR curves (Lermo and Chávez-García 1993), noting the prominent use of the Konno and Ohmachi (1998) filter in MHVSR calculation which was designed to suppress infinite peak amplitudes particularly at higher frequencies (discussed in Sect. 4.3).

Theoretically, rock sites are expected to have HVSR amplification of unity (or 1.414 according to equations in Sect. 2.3), but broadband or high-frequency amplification is often observed (e.g., Ladak et al. 2021). A relatively flat MHVSR can also result from the lack of a strong impedance contrast, even at a deep sediment site, e.g., coarse-grained soils over volcanic ash or heavily weathered (non-glaciated) rock common in Chile (Bonnefoy-Claudet et al. 2009; Leyton et al. 2013; Molnar et al. 2015) or from complex site effects (e.g., Uebayashi 2003; Di Giulio et al. 2010; Le Roux et al. 2012).

As mentioned in Sect. 4, a quick check to test the hypothesis of subsurface homogeneity is to process individual horizontal component HVSRs or directional HVSRs. If the directional HVSRs differ, it may be indicative of subsurface lateral heterogeneity. Matsushima et al. (2014) showed that over a 2D bedrock valley, the MHVSRs in two orthogonal directions are different in comparison to a 1D scenario with the same seismic parameters, MHVSR peak amplitude decreases, and *f*_0HV_ increases. Broad or plateaued peaks are often indicative of laterally variable subsurface conditions (Uebayashi 2003; Bonnefoy-Claudet et al. 2006; Ozalaybey et al. 2011; Uebayashi et al. 2012; Le Roux et al. 2012). Even in the case of very simple two-layer geology, peak frequencies can broaden over dipping resonators (Dietiker et al. 2018, 2020). Peak shapes may be asymmetrical, and shoulders may develop where resonators curve. It is not recommended to select *f*_0HV_ from plateaued curves, and a standardized procedure to select *f*_0HV_ does not exist for broad peaks. A 2D resonance pattern (when observing interpolated *f*_0HV_ on a spatial map) is common in steep-sided valleys with relatively thick sediment (Roten et al. 2006) or from edge diffracted waves due to a sloping interface. Dietiker et al. (2018) suggest that using the scaled difference of the two orthogonal spectral ratios might be a good indicator of 2D subsurface structure. They also suggest that by performing directional HVSR analysis, the orientation of subsurface structural trends can be inferred from the orientation of maximum polarization. In general, irregularly shaped peaks and large differences between orthogonal HVSRs are indicative of lateral heterogeneity.

Understanding whether observed MHVSR *f*_0HV_ variations are due to 1D or 2D/3D site effects is crucial to avoid incorrect stratigraphic interpretations. When the subsurface geology does not meet the conditions of lateral homogeneity, the spectra of the individual components contain information on the geometry and mechanical properties of the subsurface that are confused if the two components are merged (Sgattoni and Castellaro 2020). For the specific case of 2D resonance in deeply embanked valleys, the geological structure vibrates as a unique structure with a transverse (*f*_trav_) and longitudinal (*f*_long_) resonant frequency of motion. This makes 2D resonance easy to differentiate from 1D resonance because it gives two distinct peaks in the horizontal spectral components and the frequencies do not vary with space. The ratio of *f*_long_/*f*_trav_ depends on the aspect ratio of the valley; if the width is known, the depth may be determined for homogeneous valleys without any velocity gradient (Bard and Bouchon 1985). A specific combined inversion algorithm has also been proposed to account for 2D resonance frequencies (Roten and Fäh, 2007).

**Fig. 5:**
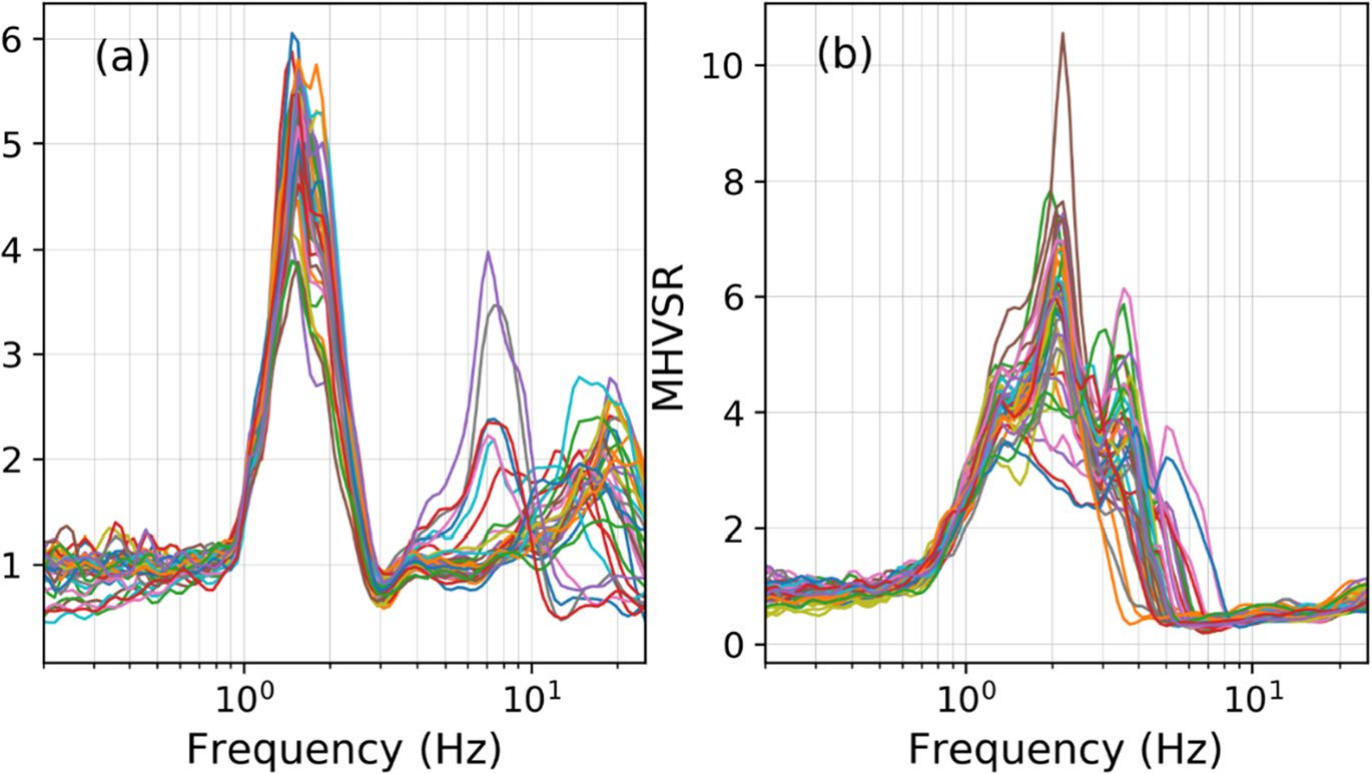
Example MHVSRs obtained during microtremor array acquisition at multiple locations (various array radius) from each array’s sensor. **a** On flat ground in Vancouver, the MHVSR peak is consistent (1.5 Hz) amongst all tested locations and indicates laterally homogeneous site geology (depth to significant impedance or resonator is consistent). A second higher frequency peak shifts in frequency (5–20 Hz) and broadness indicating variable depth and possibly geometry of near-surface resonator. **b** On sloping ground in Port Coquitlam, multiple narrow peak resonances are observed (broad to flat peak occurs when these narrow resonances have similar amplification) amongst all tested locations and indicates rapidly changing depth to significant impedance contrast (resonator) or a laterally heterogeneous site geology

**Fig. 6:**
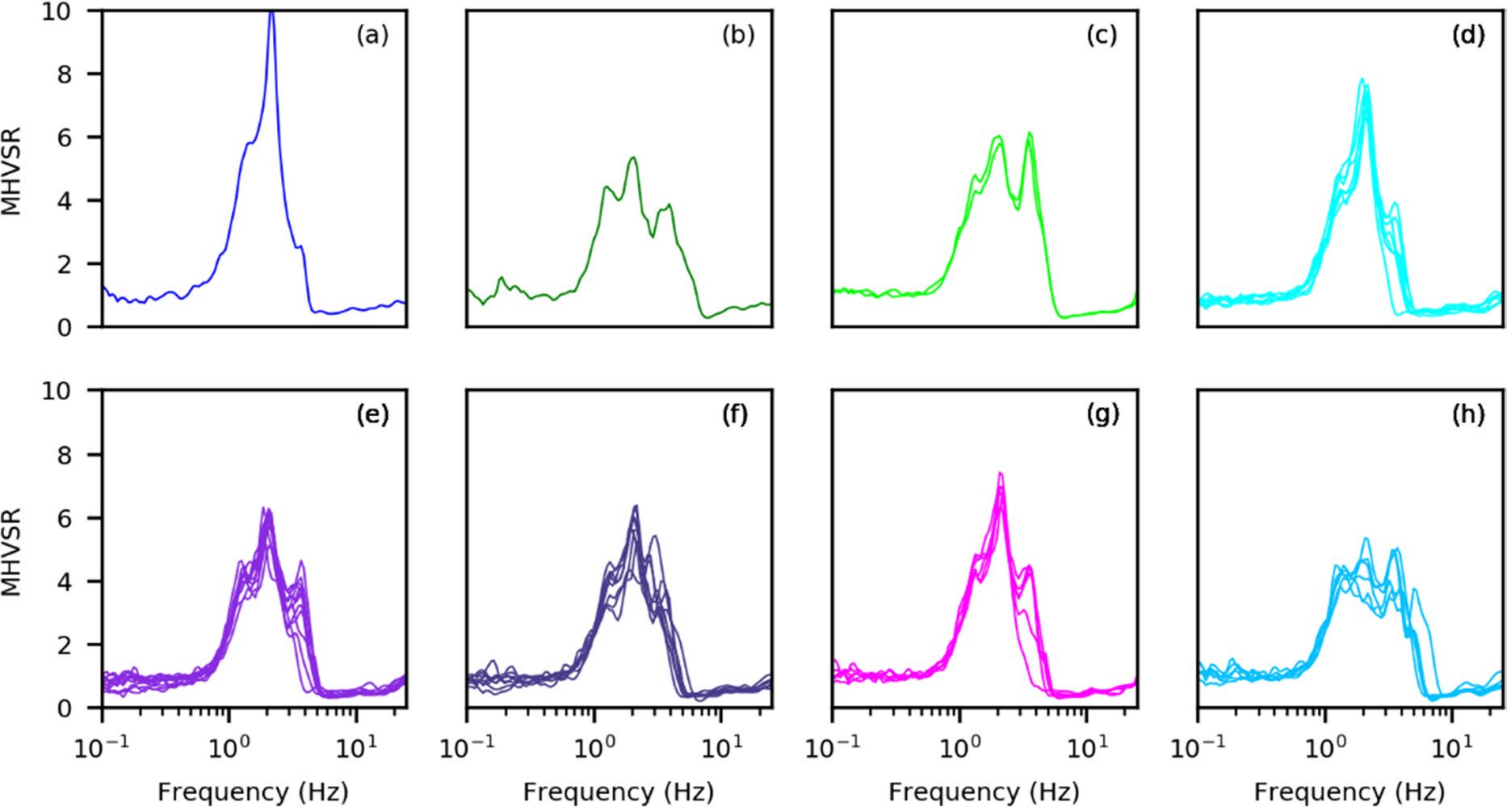
Example of eight consistent MHVSR subsets (**a**–**h**) determined by cluster analysis for the 42 MHVSRs shown in Fig. 5b

Figure 5 is provided as a summarized demonstration of MHVSR variability for 1D (Fig. 5) and 2D/3D (Fig. 5 and corresponding Fig. 6) site conditions. Figure 5 displays 42 MHVSRs obtained at a single passive seismic array site on flat ground in Vancouver, Canada; 7 seismometers were placed in a circular array of 6 different array radii of 5 to 30 m at the site. *f*_0HV_ is consistent amongst all 42 tested locations (~ 1.5 Hz), indicating homogeneous 1D site conditions. Figure 5 also displays 42 MHVSRs obtained in the same way at a different array site on sloping ground in Port Coquitlam, Canada. The MHVSRs are more variable with broadened peaks (multiple narrow resonance frequencies), indicating 2D/3D effects. Interpretation of the spatial variability of *f*_0HV_ can act as a proxy for geological site variability. A cluster analysis performed using the 42 variable MHVSRs from Fig. 5 determines up to eight subsets of the average MHVSRs (Fig. 6) confirming a highly variable spatial dependence of the MHVSR across the site. Further analysis of directional MHVSRs is required to decipher the 2D/3D effects at this site, i.e., *f*_0HV_ should not be determined from the MHVSRs in Figs. 5 and 6.

### Additional MHVSR interpretations

**Fig. 7:**
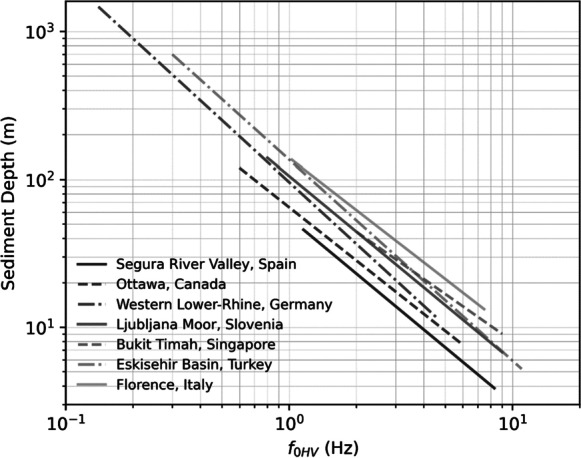
Selection of calibrated sediment depth from *f*_0HV_ relations from seven countries

With 1D interpretation of the MHVSR *f*_0HV_ as *f*_0_ (= *V*_*Save*_/*4 h*) and the assumption of flat layering in the subsurface, a common “secondary” output from MHVSR calculation is to convert *f*_0HV_ to sediment thickness or depth (*z*) (e.g., Ibs-von Seht and Wohlenberg 1999; Delgado et al. 2000; D’Amico et al. 2008; Gosar and Lenart 2010; Motazedian et al. 2011; Smith et al. 2013; Scheib et al. 2016; Tün et al. 2016; Jakica 2018; Pratt 2018; Moon et al. 2019). MHVSR results should be calibrated using detailed information about the local subsurface structure to provide reliable depth estimates. Figure 7 shows a selection of relations (Table 2) that determine sediment depth from *f*_0HV_

$$z=a{{f}_{0HV}}^{-b}$$

calibrated considering the local geology (*z* known from boreholes) in each of the seven countries. It should be noted that such a calibration and subsequent depth estimation are only accurate in flat-layered areas without a strong velocity gradient in the sediment layer (Motazedian et al. 2011). The assumption of flat-layered geology is not always correct and can negatively impact the estimation of resonator depth (e.g., Cornou et al. 2007; Guéguen et al. 2007). Where a survey objective is identification of the bedrock surface, supporting evidence is required to validate that the resonance stems from bedrock and not a sedimentary layer overlying bedrock that could give rise to similar resonance (e.g., gravel, glacial diamicton, or till). If a resonance peak resulting from a shallower resonator is misattributed to bedrock, depth to bedrock will be underestimated. Additionally, if incorrect velocities are used, or in the presence of 2D structure, estimates of depth to bedrock may be inaccurate (Roten et al. 2006; Guéguen et al. 2007).

**Table 2:** Details of selected sediment depth (*z*) from *f*_0HV_ relations shown in Fig. 7

Reference	Region	*a*	*b*	*R*^2^	*N*	f_0HV_ (Hz)	*z* (m)	Sediment type
Ibs-von Seht and Wohlenberg 1999	Western Lower Rhine, Germany	96	1.388	0.98	34	0.14–4.64	15–1600	Quaternary–Tertiary
Delgado et al. 2000	Segura River Valley, Spain	55.11	1.256	0.97	27	1.16–8.3	4.1–44.7	Holocene–Late Pleistocene
D’Amico et al. 2008	Florence, Italy	140	1.172	0.9	23	1.03–7.47	9–115	Quaternary–Pliocene
Gosar and Lenart 2010	Ljubljana Moor, Slovenia	105.53	1.25	0.58	53	0.8–9.0	5–168	Quaternary, lacustrine, and fluvial
Tun et al. 2016	Eskisehir Basin, Turkey	136	1.36	0.98	30	~ 0.3–11	~ 0–500	Quaternary–Tertiary (Vs < 800 m/s)
Motazedian et al. 2011	Ottawa, Canada	64.98	1.198	0.95	89	~ 0.6–6.0	~ 5–130	Soft glaciomarine sediments
Moon et al. 2019	Bukit Timah, Singapore	92.5	1.06	0.94	14	~ 2.0–9.0	10–45.5	Quaternary and sedimentary (Jurong) rocks

The tertiary “output” from MHVSR calculation, proposed originally but increasingly now in use, is direct use of the MHVSR curve to derive a site amplification function via inversion for a 1D earth model including elastic media properties of compressional velocity, *V*_*S*_, and density. However, the physical basis of the MHVSR (forward model) is still debated (Sect. 2), and there is no worldwide standard of MHVSR curve inversion. Wen et al. (2018) review several studies that have quantitatively interpreted the MHVSR for subsurface velocity models.

### Empirical correction of MHVSR to earthquake site amplification

Hassani et al. (2019) derived a relationship to predict EHVSR predominant frequency (*f*_d_) values, the frequency of maximum peak amplitude (not the lowest frequency peak, *f*_0HV_), from *f*_d_ values of the MHVSR calculated using response spectra. A regression analysis was conducted between *f*_d_ values obtained from earthquake and microtremor HVSRs for 70 strong motion sites in California to derive the relation:

$${log}_{10}\left({f}_{d}EHVSR\right)=\left(-0.10\pm 0.03\right)+\left(0.96\pm 0.07\right){log}_{10}\left({f}_{d}MHVSR\right).$$

They suggested that the discrepancies between the two estimates of *f*_d_, from earthquake and microtremor HVRSs, may be related to the hypothesis that most microtremor energy comes from Rayleigh waves, whereas S waves contribute the most to earthquake data. While this is an effective method to overcome discrepancies between microtremor and earthquake HVSR peak frequency values, it does not account for differences in the shape of the curve.

**Fig. 8:**
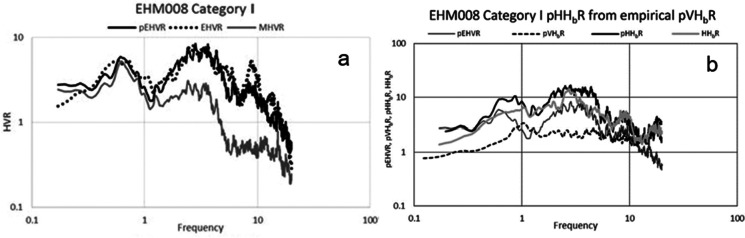
**a** Comparison of EHVR of S wave, MHVR (termed MHVSR in this paper), and pseudo-EHVSR (pEHVR) transformed from MHVSR by using empirical EMR for site EHM008 (adapted from Kawase et al. 2019; Fig. 10). The EMR correction results in a significant shift of the MHVSR amplitude towards the EHVR. **b** Comparison of pEHVR (MHVSR corrected by EMR), pHHbR (pEHVR corrected by pVHbR), and actual HHbR (SSR in this paper) for site EHM008 (adapted from Kawase et al. 2019; Fig. 13). pVHbR is the spectrum used to correct for vertical component amplification of EHVSR

**Fig. 9:**
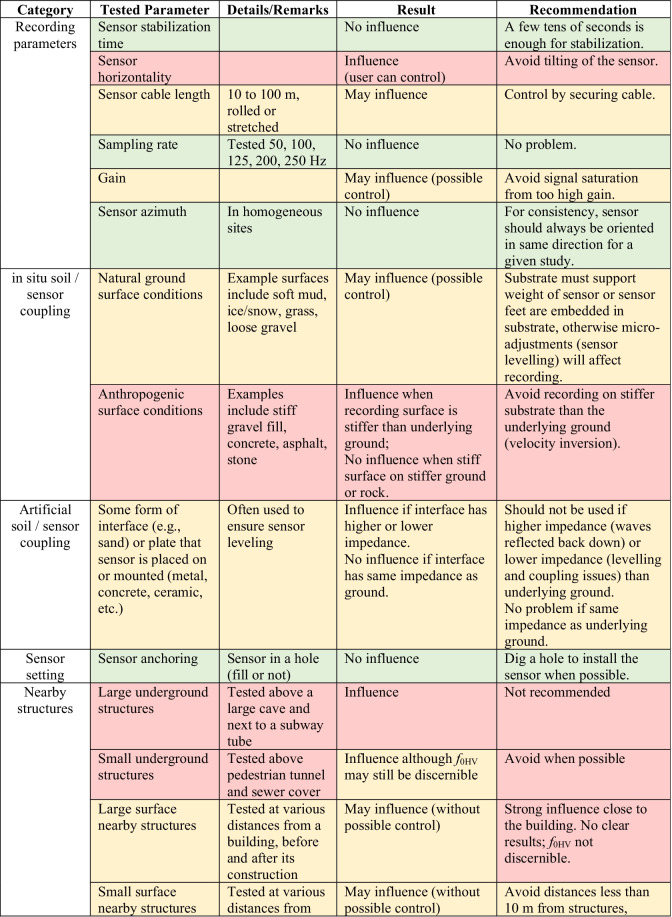
Acquisition parameters and their influence on the MHVSR. Green shading refers to parameters that have no influence, yellow shading refers to parameters that may influence (should be minimized), and red shading refers to parameters that will influence the MHVSR (should be avoided)

**Fig. 10:**
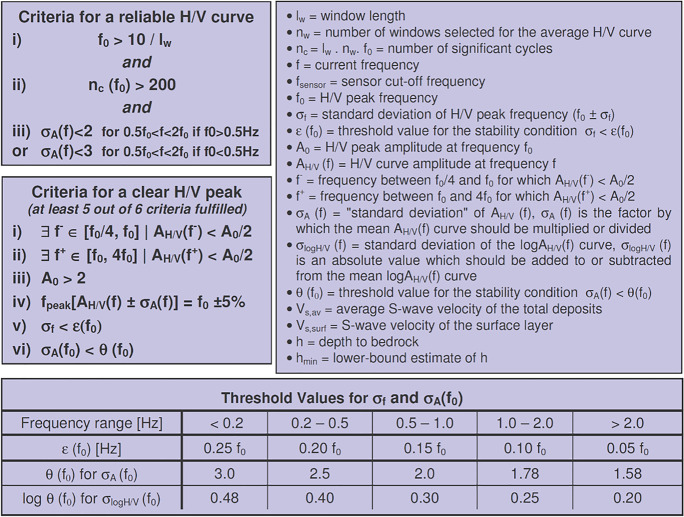
From the SESAME (2004) MHVSR guidelines, the three criteria for a reliable H/V (MHVSR) curve and six criteria for a clear H/V peak are presented

Kawase et al. (2018) developed an empirical method to correct the MHVSR shape such that it more closely approximates the EHVSR. For sites in Japan with co-located earthquake and microtremor data, the authors calculated EHVSRs and MHVSRs. They observed good agreement between the EHVSR and MHVSR up to the first peak; however, after the first peak, the EHVSR amplitude is generally greater, and higher frequency peaks (i.e., corresponding to higher modes) may be present, which are absent from the MHVSR. To correct for this, they proposed to calculate an earthquake-microtremor ratio (EMR) by taking the ratio between EHVSR and MHVSR. Before calculating the ratio, the frequency values were normalized (*f*/*f*_0_), and measurements were categorized based on *f*_0HV_. EMRs were calculated for each category by averaging the individual EMR estimates from each site within their respective category. By multiplying the MHVSR by the appropriate EMR, a closer approximation to EHVSR may be attained (Fig. 8). A recent development along the same line is due to Ito et al. (2021) in which they extend the method to apply to the Grenoble, France, basin in a different tectonic setting.

The EHVSR is a step closer to the “true” SSR answer; however, the EHVSR is still affected by amplification of vertical component motions (e.g., Lermo and Chávez-García 1993; Theodoulidis et al. 1996; Bonilla et al. 1997; Raptakis et al. 1998; 2000; Parolai et al. 2000; Rong et al. 2019; Zhu et al. 2020). Kawase et al. (2019) proposed an additional empirical correction to translate the EHVSR to SSR (Fig. 8); similar empirical corrections were suggested by Ito et al. (2020). The methodology of Kawase et al. (2019) is based on the generalized inversion technique (Andrews 1986), whereby ground motion spectra may be decomposed into source, path, and site effects. Then, utilizing a reference spectrum (i.e., free of site effects), they calculated the spectral ratio of vertical component motions to that of reference motions to determine a frequency-dependent correction. Frequency values were not normalized by *f*_0HV_, but correction spectra were calculated for the same frequency categories as EMR. The methodology was tested on sites with both earthquake and microtremor measurements, as well as suitable reference sites to calculate SSRs. The authors report an 80% success rate with regard to obtaining good correlation with SSR. Under these empirical corrections, the MHVSR can be treated as a suitable proxy for the site amplification spectrum (S wave transfer function).

## MHVSR uncertainty

### Uncertainty of *f*_0HV_

Quantifying the uncertainty inherent to *f*_0HV_ determined from microtremor (and earthquake) recordings is important as its use in ground motion prediction models and code-based seismic site classification is increasing (e.g., Zhao et al. 2006; Luzi et al. 2011; Cadet et al. 2012; Pitilakis et al. 2013; Hassani and Atkinson 2018; Harmon et al. 2019). This can be extended to estimates of *f*_0_ obtained through empirical approaches. Most often *f*_0HV_ is estimated deterministically from the mean/median MHVSR curve, which does not provide any information regarding its variability. The MHVSR curve itself is calculated in a statistical manner through the consideration of individual time windows to define a single representative mean or median curve. Given the random nature of the microtremor wavefield, variability in individual windows is expected due to unevenly distributed sources, amongst other factors.

The lowest frequency peak of the MHVSR curve (*f*_0HV_) may not be the global maximum. All peak picking algorithms search for the global maximum; thus, it is more appropriate to refer to the auto-picked peak as *f*_*gm*_, the frequency corresponding to the global maximum. The automated peak picking algorithm implemented in Geopsy (Wathelet et al. 2020) works by defining a restrictive search range [*f*_*gm,mc*_* / R*_*f*_*, f*_*gm,mc*_ * R_*f*_], where *f*_*gm,mc*_ is *f*_*gm*_ of the median curve (_*mc*_) and *R*_*f*_ is expressed as

$${R}_{f}=1.5-0.25 \frac{{f}_{gm,mc}-{f}_{min}}{{f}_{max}-{f}_{min}},$$

within which *f*_gm*,i*_ values are obtained from individual windows, where *i* denotes the individual window under consideration (Cox et al. 2020). The user defines *f*_*min*_ and *f*_*max*_ as the interval over which to calculate the MHVSR. The logic surrounding this restrictive search window is that if any window has a global peak, *f*_*gm,i*_, that is far from *f*_*gm,mc*_, then that window is likely contaminated. As opposed to removing this window from the statistical calculation of *f*_*gm*,*i*_, the algorithm searches for the local maximum within the restricted frequency range. This inclusion of contaminated windows has the potential to bias both the statistics for *f*_gm,ave_, which is the average calculated from the individual *f*_gm,*i*_ values, as well as the median curve from which *f*_*gm,mc*_ is obtained.

Cox et al. (2020) propose a frequency-domain window-rejection algorithm (FWA) that effectively removes contaminated windows from the computation of both *f*_*gm,ave*_ and the median curve. The algorithm iteratively rejects windows based on consideration of *f*_*gm,ave*_ and its corresponding standard deviation. At each iteration, these statistics are computed considering the set of windows accepted from the previous iteration. Any windows for which *f*_*gm,i*_ deviates more than *n* standard deviations from the *f*_*gm,ave*_ value determined from individual windows are rejected. The *n* value is the only user-defined parameter needed to run the FWA. Cox et al. (2020) recommended using *n* = 2.0 (i.e., two standard deviations) for most calculations. However, the exact value used for a given application should be carefully considered by the analyst to ensure reasonable time windows are not being rejected. The FWA stopping criteria involves comparison of *f*_gm,ave_ computed from individual windows and *f*_*gm,mc*_; once these values are within a certain tolerance of each other, the algorithm stops. At each iteration, the median curve and the value of *f*_*gm,mc*_ are updated. In this manner, contaminated windows are removed utilizing a statistically robust procedure and an unbiased estimate of *f*_*gm*_, and its associated standard deviation can be provided. Several methods use a log-normal distribution of MHVSR curves to determine statistics (e.g., Cox et al. 2020; Wathelet et al. 2020). Not only does this more realistically reflect the true nature of the data, but it also allows seamless transition of statistics from frequency to period.

Accounting for azimuthal variability in *f*_#HV_ is not a common procedure in spite of the fact that differences in *f*_0HV_ and its corresponding amplitude have frequently been observed in the individual horizontal components of MHVSR measurements (e.g., Guillier et al. 2006; Uebayashi et al. 2012; Matsushima et al. 2014; 2017; Ktenidou et al. 2016; Theodoulidis et al. 2018; Vantassel et al. 2018). As an extension of the FWA and statistical interpretation of MHVSR data developed by Cox et al. (2020), Cheng et al. (2020) proposed to more rigorously study MHVSR curves as a function of azimuth and statistically account for this variability in *f*_0HV_. Through the use of the FWA, a set of accepted windows is determined for each azimuth under consideration. It is also possible that for each azimuth, the number of accepted windows will differ. Thus, to avoid biasing the statistics, a weight (*w*) is applied to each *f*_0*,i*_ value prior to calculating statistics:

$$w=\frac{1}{N*I}$$

where *N* is the number of azimuths under consideration and *I* is the number of accepted windows at the azimuth under consideration.

The peak picking algorithm utilized in each of the MHVSR processing platforms discussed is restricted to identifying a single dominant peak. However, MHVSR results with several peaks (resonances), *f*_#HV_, have been observed. Accounting for the variability in these additional peaks has not been the subject of significant investigation, although there have been some observational indication of MHVRs showing different directional dependency for different peaks according to the different soil layer depths (Matsushima et al. 2019).

### Uncertainty of MHVSR curves

As described in Sect. 4, an average MHVSR curve is calculated for the recording site by averaging with time and the two horizontal components (i.e., source azimuth) derived from Aki’s (1957) proposal that microtremors are a stochastic process in time and space. It was noted that averaging of MHVSRs from all selected time windows reduces variability in the mean MHVSR curve compared to averaging of each component’s spectra with time and that the latter is considered more appropriate for use if the assumption is a diffuse wavefield. At 2D/3D sites, azimuthal variability in *f*_#HV_ occurs, and techniques are being developed to properly capture this variability (Matsushima et al. 2017; Cheng 2020).

As mentioned in Sects. 2 and 5, some advanced uses of MHVSR curves includes prediction (modelling) of the 1D *V*_*S*_ profile and/or site amplification. In addition to ongoing and vivid debates about such uses, the practitioner is reminded that (a) such uses should account for the uncertainty of MHVSR curves and the processing details (for instance, the amount of smoothing) by investigating the sensitivity of the inversion process on the MHVSR uncertainty and (b) that some parameters are not constrained at all by the inversion process. For example, the damping profile, needed for deriving the forward amplification function from the velocity profile, cannot presently be obtained from the MHVSR curve. Another example is the derivation of ellipticity from special processing of the MHVSR curve and its inversion in terms of the velocity profile, which requires some anchoring *V*_*S*_ value at some depth: as shown in Scherbaum et al. (2003) and illustrated in Fig. 1 of Hobiger et al. (2013), Rayleigh wave ellipticity curves remain unchanged when velocity and depth are scaled by the same factor *k* (i.e., *V*_*S*_(*z*) and *k*.*V*_*S*_(*k*.*z*), and their inversion should be constrained by an independent estimate of *V*_*S*_ at some depth, for instance, at surface (the same is true for the inversion of the S wave transfer function).

## Conclusions

This paper began with splitting the greatest challenges to an international standardization of the MHVSR method into the physical basis of the MHVSR and its underlying wavefield composition (the *what*) and recommendations of MHVSR acquisition and analysis including its interpretation and uncertainty assessment (the *how*). In conclusion, we split summary of aspects of these two great challenges to MHVSR method standardization into those that are known or undebated from those that are still debated or ongoing research.

The potential and therefore use of the MHVSR method to provide a reliable estimate (proxy) of the site fundamental frequency or period have been known for approximately 3 decades with debate tapering off about 2 decades ago. The act of retrieving *f*_0HV_ and secondary peaks (*f*_#HV_) is therefore not debated, but *how* it is accomplished still is. This paper summarized a selection of literature documenting the increasing automation and innovation of quantitative criteria towards retrieval of *f*_#HV_ which continues to be an area of active research. Early on, *f*_0HV_ was directly used to determine engineering bedrock depth (first instance of geologic rock under soils) but later literature reported: (1) that this is a resonator depth and (2) development of local calibrations for converting *f*_0HV_ to sediment depth. The determination of *f*_0HV_ at a particular location, or the mapping of its spatial variability around a site or area (regional microzonation), and its conversion to sediment thickness have always been and still are the predominant uses of the MHVSR method.

In the 2000s, the first consortium effort in recommendations for MHVSR field measurements, processing, and interpretation occurred (the SESAME project; Bard et al. 2008). In a general sense, it is no longer debated how to perform MHVSR acquisition and analysis; many practitioners have been doing so and training others for decades. The increasing commercial production in seismic equipment and associated software for MHVSR acquisition and analysis (and passive seismology in general) is a form of attestation to this undebated knowledge. However, details of *how* MHVSR acquisition and analysis are accomplished are still debated. The most debated aspects amongst this paper’s authors included equipment recommendations (various seismic systems are available to us and selected depending first on the site conditions and then on the suitability of operation given the project or environment; the latter is worth conveying to the untrained but can also convey unintended preference of particular brand names which is avoided here without a recent and comprehensive blind test or benchmark experiment), field acquisition recommendations (difficulties due to unlimited worldwide conditions or “exceptions to the rule”), and the rapid outdatedness of discussing particular analysis techniques and software (areas of current research).

There have been significant and important advancements into the physical basis of the MHVSR curve (the *what*) and its underlying microtremor wavefield composition (the appropriate forward model) throughout the past two decades. A total/diffuse wavefield approach is well suited towards a worldwide standardization, allowing the data itself to determine wavefield contributions rather than our assumptions. Hence, the current most controversial aspect of the MHVSR method is our ability to model (invert) the MHVSR curve for 1D layered earth models (simplified to *V*_*S*_ depth profiles) and whether we should even be doing so without some form of correction/calibration particularly at frequencies above *f*_0HV_. A comprehensive and/or international benchmark test of the multitude of available equipment types and their ability to provide consistently “the same” MHVSR curve is necessary towards the inversion of MHVSR curves for subsurface models.

## Data Availability

Not applicable.

## Appendix 1

﻿A table of acquisition parameters and their influence on the MHVSR is provided in Fig. 9, updated from Koller et al. (2004). These types of metadata associated with each microtremor recording are important to include in field notes and refer to during MHVSR calculation and interpretation.

## Appendix 2

Many software packages are available now to process and analyze MHVSR measurements. Examples include Grilla, Geopsy, ModelHVSR, OpenHVSR, and HVSRPy. Most of these software suites provide the user with the ability to pre-condition (filter) time series data, if necessary. Time window selection may be accomplished manually, directly from the screen, or through the use of a time-domain anti-trigger algorithm. Once the time windows have been transformed to the frequency domain, the user has the option to smooth the spectra and decide how to combine the horizontal components to define a representative horizontal spectrum or opt to calculate two separate ratios and treat each component independently. Below is a review of currently available MHVSR processing software. Although with advancements ongoing, improvements to these software platforms may make some of the descriptions obsolete; this Appendix section is meant to provide readers with a snapshot of the current state of available MHVSR processing.

Grilla is a proprietary software to analyze Tromino® recordings (http://moho.world/en/) (Castellaro and Mulargia 2009a, b) and accepts several other non-proprietary input formats. It has the capability to perform spectral analyses, as well as time-dependent and direction-dependent MHVSR analyses.

Geopsy (Wathelet et al. 2020) is an open-source software developed from the SESAME project. It accepts a variety of different input file formats. Both single measurement and batch processing of MHVSRs are supported. Geopsy automatically provides a value of *f*_0_ from the mean MHVSR curve (deterministically), as well as from the individual windows (statistically) that can be adjusted by the user such as to delete spurious individual MHVSR curves. Geopsy provides tools to compute the MHVSR as a function of azimuth (rotate) and complete time frequency analysis, which can aid in wave composition analyses (Fäh et al. 2009). Geopsy has modules to forward model the ellipticity curve and the SH-transfer function for layered earth models.

ModelHVSR (Herak 2008) consists of several MATLAB-based modules and algorithms. It does not have the capability to deal with raw time series data, so the input data must already be transformed to the frequency domain. AVG_HVSR calculates the average MHVSR from any number of observed MHVSRs. The input to this module is the spectral data, and an option is available to apply spectral smoothing or alternatively to use Landweber filtering on the unsmoothed spectra. ModelHVSR can forward model the theoretical S wave transfer function of a layered viscoelastic model for vertically incident S waves (horizontal response) and the theoretical P wave amplification spectrum for vertically incident P waves and calculates the MHVSR as the ratio between these two quantities.

OpenHVSR (Bignardi et al. 2018) is a MATLAB-based program capable of generating and displaying MHVSR curves from raw field data. One of its strengths is the ability to handle large volumes of data and spatially correlate different forms of informative data content. All data are loaded into the same environment, which facilitates batch processing of MHVSRs, allowing easy implementation of sensitivity analyses to test the choice of a specific set of processing parameters on the output MHVSRs. An interesting feature of this software is the implementation of directional analysis of spectral ratios to check whether the data contain non-isotropic components. Spatial map products can be produced of resonance frequency values, amplitude at resonance peaks, and preferential direction of *f*_0_. OpenHVSR utilizes the same forward modelling strategy as ModelHVSR; however, it also provides the ability to simulate the surface wave contribution using the modelling routine of Lunedei and Albarello (2010).

HVSRPy is an open-source Python package for HVSR processing that was developed recently by Vantassel (2020) to implement new statistical MHVSR processing techniques proposed by Cox et al. (2020) and Cheng et al. (2020; 2021). Several features of HVSRPy make it unique from other available software. These novel functionalities include a frequency-domain window-rejection algorithm (FWA), which allows for the automatic rejection of contaminated time windows in the frequency domain, the ability to incorporate azimuthal variability into the statistical representation of *f*_0_, and the use of log-normal statistics, which allow for consistent representation of uncertainty, regardless of whether *f*_0HV_ or its reciprocal *T*_0_ is the desired parameter of interest. Additionally, HVSRPy allows for calculating an unbiased, statistical representation of *f*_0_ or *T*_0_ from numerous, spatially distributed MHVSR measurements using Voronoi tessellations for spatial de-clustering. Batch-style processing is simple and easy to implement using HVSRPy. For those who wish to quickly process a single MHVSR record without the need to deal with Python code, a web-based application called HVSRweb has also been developed (Vantassel et al. 2018). HVSRweb currently has all of the functionality of HVSRPy except for the ability to calculate statistics from spatially distributed MHVSR measurements. HVSRweb can be accessed at https://hvsrweb.designsafe-ci.org/.

The HVSR IRIS station toolbox (Bahavar et al. 2020) corresponds to the Incorporated Research Institutions for Seismology (IRIS) Data Management Center’s (DMC’s) openly available HVSR station toolbox. The platform includes a variety of ways to compute the spectral ratio for ambient seismic noise by providing different averaging routines. The options range from the simple average of spectral ratios to the ratio of spectral averages. Computations take advantage of the available power spectral density estimates of ambient noise for the seismic stations, and they can be used to estimate the predominant frequency of the many three-component seismic stations available from the IRIS DMC.
